# Enhancement of CURB65 score with proadrenomedullin (CURB65-A) for outcome prediction in lower respiratory tract infections: Derivation of a clinical algorithm

**DOI:** 10.1186/1471-2334-11-112

**Published:** 2011-05-03

**Authors:** Werner C Albrich, Frank Dusemund, Kristina Rüegger, Mirjam Christ-Crain, Werner Zimmerli, Thomas Bregenzer, Sarosh Irani, Ulrich Buergi, Barbara Reutlinger, Beat Mueller, Philipp Schuetz

**Affiliations:** 1 Medical University Department of the University of Basel, Kantonsspital Aarau, Switzerland; 2 Department of Internal Medicine, Division of Endocrinology, Diabetes and Clinical Nutrition, University Hospital Basel, Switzerland; 3 Basel University Medical Clinic, Department of Internal Medicine, Kantonsspital Liestal, Switzerland; 4 Department of Infectious Diseases, Kantonsspital Aarau, Switzerland; 5 Department of Pulmonary Medicine, Kantonsspital Aarau, Switzerland; 6 Department of Emergency Medicine, Kantonsspital Aarau, Switzerland; 7 Department of Nursing, Kantonsspital Aarau, Switzerland; 8 Harvard School of Public Health, Boston (MA), USA

## Abstract

**Background:**

Proadrenomedullin (ProADM) confers additional prognostic information to established clinical risk scores in lower respiratory tract infections (LRTI). We aimed to derive a practical algorithm combining the CURB65 score with ProADM-levels in patients with community-acquired pneumonia (CAP) and non-CAP-LRTI.

**Methods:**

We used data of 1359 patients with LRTI enrolled in a multicenter study. We chose two ProADM cut-off values by assessing the association between ProADM levels and the risk of adverse events and mortality. A composite score (CURB65-A) was created combining CURB65 classes with ProADM cut-offs to further risk-stratify patients.

**Results:**

CURB65 and ProADM predicted both adverse events and mortality similarly well in CAP and non-CAP-LRTI. The combined CURB65-A risk score provided better prediction of death and adverse events than the CURB65 score in the entire cohort and in CAP and non-CAP-LRTI patients. Within each CURB65 class, higher ProADM-levels were associated with an increased risk of adverse events and mortality. Overall, risk of adverse events (3.9%) and mortality (0.65%) was low for patients with CURB65 score 0-1 and ProADM ≤0.75 nmol/l (CURB65-A risk class I); intermediate (8.6% and 2.6%, respectively) for patients with CURB65 score of 2 and ProADM ≤1.5 nmol/l or CURB classes 0-1 and ProADM levels between 0.75-1.5 nmol/L (CURB65-A risk class II), and high (21.6% and 9.8%, respectively) for all other patients (CURB65-A risk class III). If outpatient treatment was recommended for CURB65-A risk class I and short hospitalization for CURB65-A risk class II, 17.9% and 40.8% of 1217 hospitalized patients could have received ambulatory treatment or a short hospitalization, respectively.

**Conclusions:**

The new CURB65-A risk score combining CURB65 risk classes with ProADM cut-off values accurately predicts adverse events and mortality in patients with CAP and non-CAP-LRTI. Additional prospective cohort or intervention studies need to validate this score and demonstrate its safety and efficacy for the management of patients with LRTI.

**Trial Registration:**

Procalcitonin-guided antibiotic therapy and hospitalisation in patients with lower respiratory tract infections: the prohosp study; isrctn.org **Identifier**: ISRCTN: ISRCTN95122877

## Background

Lower respiratory tract infections (LRTIs) comprising community-acquired pneumonia (CAP), exacerbation of chronic obstructive pulmonary disease (COPD) and acute bronchitis, are the most important infectious causes of death in industrialized countries [1]. In the management of CAP, assessment of disease severity and prediction of outcome is essential for a rational allocation of health care resources and for guiding therapeutic options. For this purpose, different risk assessment tools have been developed. The CURB65 is a widely used risk assessment tool [2,3]. It relies on only five predictors, is relatively easy to memorize and calculate, and demonstrates better practicability than other scores [4,5]. However, it conceptually is a static score and lacks information on the host's inflammatory response. The score was validated to predict mortality only and has not been proven to predict other CAP-associated adverse outcomes. CURB65 has been validated in many studies for CAP patients, and to a lesser extent in other non-CAP-LRTI patients [6,7]. It provides only limited discriminatory power with moderate sensitivity and specificity. This potentially leads to hospitalization of low-risk patients where outpatient treatment would be preferable, and conversely, to outpatient treatment of high-risk patients [4]. Thus, there is increasing interest in new risk factors and biomarkers that confer additional prognostic information [8,9].

Several candidate hormones have been proposed, among which proadrenomedullin (ProADM) proved to be the most promising in patients with sepsis [10-12], CAP [13-15] and other LRTIs [13,16]. ProADM belongs to the calcitonin peptide superfamily and emerges from the CALC V gene with distinct molecular regulation and various effects, including potent vasodilatation, immune modulation and bactericidal activity [16,17]. ProADM is a "hormokine", characterized by a hormone-like behavior in non-inflammatory conditions when it is produced only by endocrine cells; and a cytokine-like behavior in septic conditions when it is ubiquitously hyper-expressed [18,19]. We recently validated the prognostic performance of ProADM in a large cohort of LRTI patients from a multicenter study [13]. A precise clinical algorithm for future routine use, however, remains to be established. The aim of this analysis was, therefore, to derive a risk score based on CURB65 classes enhanced by ProADM cut-off ranges for improved prognostic assessment in CAP and non-CAP-LRTIs as a basis for future intervention studies.

## Methods

### Study Design and Setting

From October 2006 to March 2008, 1359 consecutive patients with presumed LRTI from six Swiss hospitals were included in the randomized controlled ProHOSP study to determine the safety of procalcitonin-guided antimicrobial therapy [20,21]. As a predefined secondary endpoint, we investigated the prognostic potential of ProADM and other biomarkers.

### Selection of participants

Adult patients (>18 years) with LRTI as principal diagnosis on admission were eligible as previously described [20]. CAP was defined as a new infiltrate on chest radiograph; COPD by spirometric criteria, according to the Global Initiative for Chronic Obstructive Lung Disease (GOLD) guidelines; acute bronchitis by LRTI in the absence of an underlying lung disease or focal clinical and radiological findings [22-25]. For all patients, the CURB65 was calculated on admission [3]. The study protocol was approved by all local ethical committees, and written informed consent was obtained from all participants.

### Methods of ProADM measurement

ProADM was collected on admission (day 0) and on days 3, 5 and 7 and batch-measured in a blinded fashion with a sandwich immunoassay with an analytical detection limits of 0.08 nmol/L as described elsewhere [26].

### Study endpoints

The primary endpoint for this analysis was a composite of adverse events defined as death of any cause, intensive care unit (ICU) admission, or any disease specific complications (i.e, persistence or development of pneumonia, lung abscess, empyema, and acute respiratory distress syndrome) within 30 days after enrollment. The secondary endpoint was all-cause 30-day mortality. We displayed internationally-accepted severity criteria according to the 2001 American Thoracic Society recommendations on a study web-site [24]. In brief, we recommended that patients should be assessed for need of transfer to the ICU if they had severe CAP [27], defined as the presence of either one of two major criteria (need for mechanical ventilation, septic shock), the presence of two of three minor criteria (systolic blood pressure <90 mmHg, multilobar disease, PaO2/FIO2 ratio <250), or more than 2 CURB65 points [3]. For COPD patients, severity was defined based on modified ERS guidelines [28], including severe acidosis or respiratory failure (pH<7.3, pO2<6.7 kPa, pCO2>9.3 kPa), no response to initial treatment in the emergency department or worsening mental status (confusion, coma) despite adequate therapy. Yet, these recommendations were not mandatory and the final decision for ICU transfer was a clinical decision made by the attending physician in charge who also incorporated the social situation, patient's requests and the hospital capacity into the decision-making. We also assessed outpatient treatment within the ProHOSP study, which was defined as discharge from the hospital or emergency department after 1 day or less.

Standardized outcome assessment was performed during hospital stay and by structured phone interviews on day 30 by trained medical students. It was monitored by an independent Data Safety and Monitoring Board consisting of 3 specialists in pulmonary medicine, infectious diseases and intensive care medicine as part of the study protocol [21]. If the patient indicated any complication during or following hospital discharge, or was unable to give adequate information, respectively, the interviewer contacted the treating physician or the hospital to obtain further information or the discharge letter.

### Statistical Analysis

The primary analysis population contained all 1359 patients with presumed LRTI from the ProHOSP study. We also calculated results for CAP and non-CAP-LRTI patients separately. As described previously [13], we used imputations to deal with missing CURB65 covariates and ProADM values. The imputation dataset consisted of all 1359 ProHOSP patients and the following variables: all covariates included in the derivation of the pneumonia severity index (PSI) or CURB65 scores, biomarker values on day 0, 3, 5, and 7, randomization arm, final diagnosis, total antibiotic exposure, length of hospital stay as well as death, ICU admission, complication, or disease recurrence within 30 days of randomization. Outcomes were also included in the imputation to avoid bias. We then used average values over five imputed datasets for the final analysis.

First, we visualized the (smoothed) association between ProADM levels on admission and the risk of serious complications or mortality, respectively, based on a generalized additive model for each CURB65 risk category (CURB65 0-1; 2; 3-5). We tested whether ProADM improves the performance of the CURB65 score by comparing ROC curves of the joint logistic regression of ProADM and the CURB65 to ROC curves limited to the CURB65 only. We then calculated separate logistic regression models for serious complications and death with log-transformed ProADM-levels as the sole covariate within CURB65 risk categories. Predicted and observed probabilities for both endpoints were displayed within deciles of ProADM and goodness-of-fit assessed with the Hosmer-Lemeshow test. Based on these analyses, we chose two ProADM cut-off values which meaningfully separated patients as low or high risk. A composite score (CURB65-A) was created combining CURB65 class with ProADM cut-offs to further stratify patients in low (CURB65-A risk class I), intermediate (CURB65-A risk class II) and high risk (CURB65-A risk class III). We then investigated the clinical usefulness of these cut-off values and risk classes by calculating observed risks based on the ProHOSP study sample.

Finally, we investigated the potential utility of the CURB65-A score for triage decisions.

All testing was two-tailed and p values less than 0.05 were considered to indicate statistical significance. All calculations were performed using STATA 9.2 (Stata Corp, College Station, Texas).

## Results

### Patient population

A total of 1359 patients (58% males, median age 73 years) with initial suspicion of LRTI were included (Table 1). Of these, 1304 had a definite diagnosis of LRTI including 925 patients with CAP, 228 patients with exacerbation of COPD, and 151 patients with acute bronchitis. At least one comorbidity was observed in >70% of patients.

**Table 1:** Baseline characteristics

Characteristics	All patients (n = 1359)
**Demographic characteristics**	
Age (years)*	73 (59-82)
Sex (male) - no. (%)	782 (57.5)
**Coexisting illnesses **- no. (%)	
Coronary heart disease	282 (20.8)
Cerebrovascular disease	110 (8.1)
Renal dysfunction	302 (22.2)
COPD	533 (39.2)
Neoplastic disease	167 (12.3)
Diabetes	231 (17.0)
Any coexisting illness	963 (70.9)
**Clinical history **- no. (%)	
Cough	1164 (88.7)
Expectorations	678 (50.9)
Dyspnea	1009 (77.0)
Fever	782 (57.9)
Chills	362 (32.0)
**Clinical findings**	
Confusion - no. (%)	84 (6.8)
Respiratory rate (breaths/min)*	20 (16-25)
Systolic blood pressure (mmHg)*	134 (120-150)
Heart rate (beats/minute)*	93 (80-106)
Body temperature (C°)*	37.8 (37.0-38.6)
Rales - no. (%)	832 (64.1)
**Final diagnosis - no. (%)**	
Community-acquired pneumonia (CAP)	925 (68.1)
Exacerbation of COPD	228 (16.8)
Bronchitis	151 (11.1)
Other final diagnosis	55 (4.0)

*COPD*, chronic obstructive pulmonary disease; *CAP*, community-acquired pneumonia; *PSI*, pneumonia severity index; higher PSI class refers to higher risk for mortality; *expressed as median (Interquartile range, IQR);

Note: there was no statistical difference in baseline characteristic between both groups.

### Performance of CURB65 and ProADM overall and in CAP and non-CAP-LRTI

Overall, 12.2% of patients experienced an adverse event within 30 days of enrollment, including death (4.9%) and ICU admission (7.6%). Disease-specific complications - all of them empyema - occurred in 2.4% and only in CAP patients. CURB65 and initial ProADM showed a similar performance in the overall cohort, and in patients with CAP and non-CAP-LRTI (Table 2).

**Table 2:** Outcomes and performance of risk scores and ProADM overall and in CAP and non-CAP-LRTI

Parameters	All patients (n = 1359)	CAP (n = 925)	Non-CAP (n = 379)	P*
**30 days outcomes**				
Outpatient treatment, no (%)	142 (10%)	81 (9%)	61 (16%)	*<0.001*
Length of hospital stay, median (IQR)	8 (4-12)	8 (5-12)	7 (3-11)	*<0.001*
All cause mortality, no (%)	67 (4.9%)	50 (5.4%)	10 (2.6%)	*0.03*
ICU admission, no (%)	103 (7.6%)	83 (9.0%)	15 (4.0%)	*0.002*
Empyema, no (%)	31 (2.4%)	31 (3.3%)	0	*<0.001*
Any adverse events, no (%)	170 (12.2%)	134 (14.5%)	24 (6.3%)	*<0.001*
				
**CURB65 score**				
CURB65 points, median (IQR)	2 (1-2)	2 (1-2)	1 (1-2)	*0.002*
**CURB65 risk classes**				*0.001*
CURB65 0-1, no (%)	659 (48.5%)	427 (46.2%)	211 (55.7%)	
CURB65 2, no (%)	434 (31.9%)	296 (32.0%)	114 (30.1%)	
CURB65 3-5, no (%)	266 (19.6%)	202 (21.8%)	54 (14.2%)	
AUC of CURB65 risk classes for adverse events (95%CI)	0.65 (0.61-0.69)	0.64 (0.59-0.65)	0.68 (0.57-0.78)	
AUC of CURB65 risk classes for mortality (95%CI)	0.73 (0.68-0.75)	0.72 (0.66-0.77)	0.74 (0.62-0.86)	
				
**Initial proADM level (day 0)**				
ProADM, median (IQR) (nmol/l)	1.1 (0.7-1.7)	1.2 (0.8-1.9)	0.9 (0.6-1.3)	*<0.001*
**ProADM categories**				*<0.001*
ProADM <0.75 nmol/l, no (%)	353 (26.0%5)	194 (21.0%)	145 (38.3%)	
ProADM: 0.75 - 1.5 nmol/l, no (%)	588 (43.3%)	400 (43.2%)	166 (43.8%)	
ProADM >1.5 nmol/l, no (%)	418 (30.8%)	331 (35.8%)	68 (17.9%)	
AUC of proADM for adverse events (95%CI)	0.73 (0.69-0.78)	0.71 (0.67-0.78)	0.76 (0.66-0.85)	
AUC of proADM for mortality (95%CI)	0.79 (0.73-0.85)	0.76 (0.68-0.83)	0.88 (0.80-0.94)	

AUC Area under the ROC curve; PSI Pneumonia Severity Index; CURB65 confusion, uremia, respiratory rate, blood pressure, age 65 years or greater; p value relates to difference between CAP and non-CAP-LRTI; of note, some patients had more than one adverse event, thus the numbers may not sum up to 100%. * Comparison CAP vs. Non-CAP

### Addition of ProADM to CURB65 score for mortality and adverse event prediction

When adding ProADM to the CURB65 score in a joint logistic regression model, the combined model showed a significant improvement of the CURB65 score alone for mortality prediction and adverse event prediction in ROC statistics. The AUC of the combined model in the overall cohort was 0.81 (95%CI: 0.77-0.86) for mortality prediction and 0.74 (95%CI: 0.70-0.79) for adverse event prediction (p < 0.0001 compared to CURB65 score for both comparisons). For CAP patients, the respective AUCs were 0.80 (0.73-0.86; p < 0.00001) and 0.73 (0.68-0.78; p < 0.00001); for non-CAP-LRTI patients the AUCs were 0.88 (0.81-0.95; p < 0.05) and 0.76 (0.67-0.85; p < 0.01).

### Predicted risks of ProADM and optimal cut-off values

**Figure 1:**
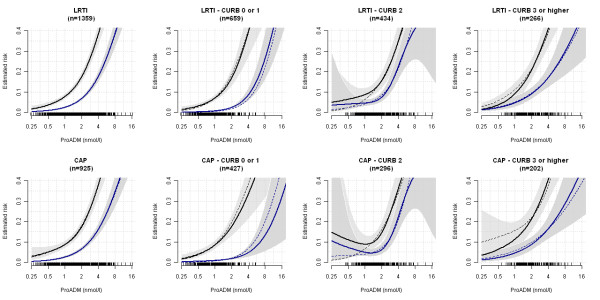
**Estimated association between initial ProADM values and the risk of adverse events (A) and death (B)**. Estimated association between initial ProADM values and the risk of adverse events (upper black line) and death (lower blue line). Estimates are based on generalized additive models and shaded gray regions correspond to (point-wise) 95% confidence intervals. The rugs at the bottom of the plots display the distribution of the biomarker. Solid lines (and confidence intervals) based on imputed data, dashed lines based on complete-case analysis.

**Figure 2:**
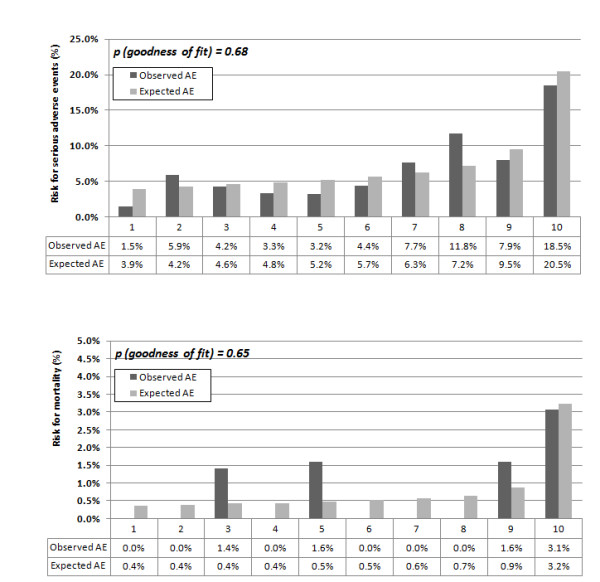
**Observed and expected adverse events (A) and mortality (B) within CURB65 class 0/1 (low risk patients)**. Observed and expected adverse events and mortality within CURB65 class 0/1 (low risk patients). A. Calibration of initial ProADM deciles and adverse events. B. Calibration of ProADM deciles and mortality.

**Figure 3:**
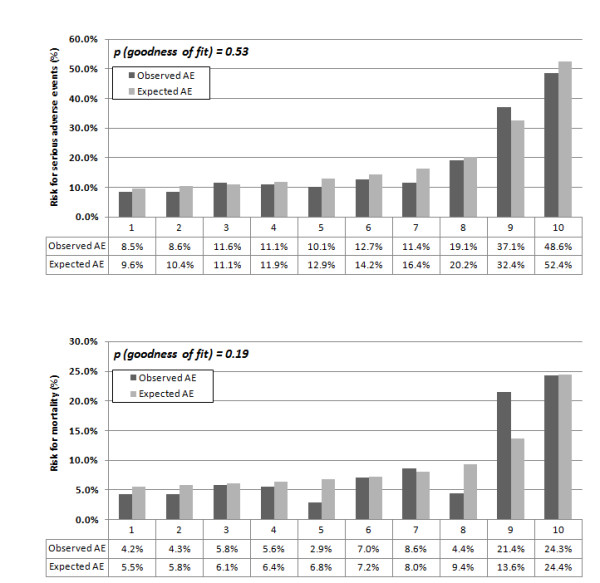
**Observed and expected adverse events (A) and mortality (B) within CURB65 class 3-5 (high risk patients)**. Observed and expected adverse events and mortality within CURB65 class 3-5 (high risk patients). A. Calibration of ProADM deciles and adverse events. B. Calibration of initial ProADM deciles and mortality.

In Figure 1, the estimated smoothed associations between admission ProADM levels and predicted adverse events risks (black line) and predicted mortality risk (blue line) are displayed. Hosmer-Lemeshow goodness of fit test showed no evidence for miscalibration (p > 0.05 for all calculations) in the plots based on a logistic regression model depicting adverse events and mortality for ProADM within CURB65 groups. Within low risk CURB65 classes 0 and 1, patients in the lowest three ProADM deciles (approximately corresponding to a ProADM level of <0.75 nmol/L), the probability for adverse events was <5% and the probability for mortality was <0.5%, but increased to >20% and >3% in the highest decile (Figure 2). Within *a priori *high-risk CURB65 classes 3-5, the risk for adverse events and mortality increased to >20% and >9% in the highest three deciles of ProADM levels (approximately corresponding to a ProADM level of >1.5 nmol/L) (Figure 3).

### ProADM enhanced CURB65 risk score - CURB65-A

**Figure 4:**
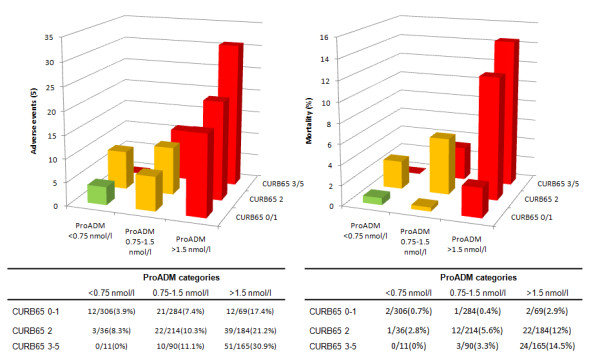
**Combination of CURB65 risk classes and ProADM tertiles in all LRTI patients**. Combination of CURB65 risk classes and initial ProADM tertiles in all LRTI patients. A. Adverse events in CURB65 and ProADM categories. B. Mortality in CURB65 and ProADM categories. Risk class I = green, risk class II = orange, risk class III = red.

**Figure 5:**
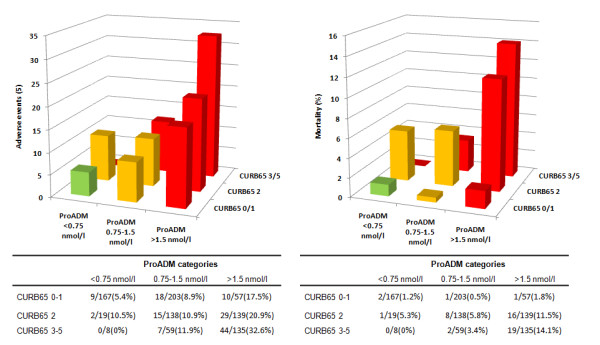
**Combination of CURB65 risk classes and ProADM tertiles in CAP patients**. Combination of CURB65 risk classes and initial ProADM tertiles in CAP patients. A. Adverse events in CURB65 and ProADM categories. B. Mortality in CURB65 and ProADM categories. Risk class I = green, risk class II = orange, risk class III = red.

We assessed observed risks for adverse outcome and mortality within all patients and within CAP patients only based on the two ProADM cut offs and the three *a priori *CURB65 risk groups (Table 3 Figures 4 &5). Thereby, patients in lowest CURB65 groups and with ProADM levels of <0.75 nmol/L had very low risks for both adverse events and mortality (CURB65-A risk class I). Conversely, patients in the highest CURB65 group and in the highest ProADM groups (CURB65-A risk class III) had high risk for adverse events and for mortality. Only 11 patients had ProADM levels of <0.75 nmol/L but were in the highest CURB65 classes 3-5 and none of them had an adverse outcome. Finally, patients within CURB65 class 2 but with ProADM levels of <1.5 nmol/L or CURB classes 0-1 and ProADM levels between 0.75-1.5 nmol/L had intermediate risks (CURB65-A risk class II). In patients with high CURB65 classes, increasing ProADM levels indicated increasing risk of adverse events and of mortality (p for trend 0.03 and p = 0.09, respectively, in CURB65 class 2; and p for trend <0.001 and p = 0.02, respectively, in CURB65 classes 3-5). In low-risk patients (CURB65 classes 0-1) increasing ProADM levels provided no additional information on mortality risk (p for trend: 0.98), but indicated significantly increased risk of adverse events (p for trend: 0.008).

**Table 3:** Observed mortality rate according to risk class CURB65-A (based on ProADM and CURB65)

	All LRTI patients (1359)			CAP patients (n = 925)		
	**n**	**Adverse events (95%CI)**	**Mortality** **(95%CI)**	**n**	**Adverse events (95%CI)**	**Mortality** **(95%CI)**

**CURB65 risk classes**						
**CURB65 0-1**	659	6.83 (4.9-8.76)	0.76 (0.09-1.42)	427	8.67 (5.99-11.34)	0.94 (0.02-1.85)
**CURB65 2**	434	14.75 (11.4-18.1)	8.06 (5.49-10.64)	296	15.54 (11.39-19.69)	8.45 (5.26-11.63)
**CURB65 3-5**	266	22.93 (17.85-28.02)	10.15 (6.5-13.8)	202	25.25 (19.21-31.29)	10.4 (6.15-14.64)
						

**ProADM categories**						

**ProADM <0.75 nmol/l**	353	4.25 (2.13-6.36)	0.85 (-0.11-1.81)	194	5.67 (2.39-8.95)	1.55 (-0.21-3.3)

**ProADM 0.75 - 1.5 nmol/l**	588	9.01 (6.69-11.34)	2.72 (1.4-4.04)	400	10 (7.05-12.95)	2.75 (1.14-4.36)

**ProADM >1.5 nmol/l**	418	24.4 (20.27-28.54)	11.48 (8.41-14.55)	331	25.08 (20.38-29.77)	10.88 (7.5-14.25)

						

**CURB65-A**						

**Risk class 1**	306	3.92 (1.73-6.11)	0.65 (0.25-1.56)	167	5.39 (1.93-8.85)	1.2 (0.47-2.86)

**Risk class 2**	534	8.61 (6.23-11)	2.62 (1.26-3.98)	360	9.72 (6.65-12.8)	2.78 (1.07-4.48)

**Risk class 3**	519	21.58 (18.03-25.13)	9.83 (7.26-12.4)	398	22.61 (18.49-26.74)	9.55 (6.65-12.45)

						

**Total**	1359	12.51 (10.75-14.27)	4.93 (3.78-6.08)	925	14.49 (12.21-16.76)	5.41 (3.95-6.87)

### Potential effects on triage decisions

**Figure 6:**
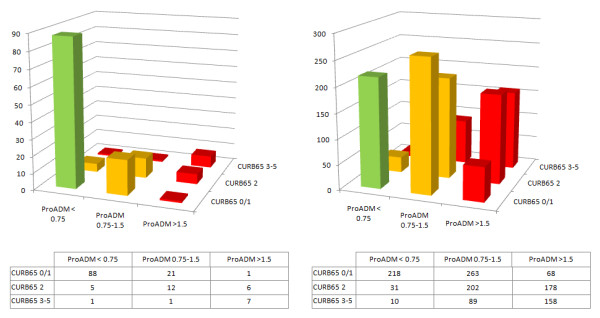
**Risk classification stratified for outpatients (A) and inpatients (B)**. Risk classification stratified for outpatients (A) and inpatients (B) according to CURB65-A. Risk class I = green, risk class II = orange, risk class III = red.

We further assessed potential impact of this new risk score on in- and outpatient treatment. Of 142 patients who were treated as outpatients, ambulatory treatment would indeed be recommended for 88 (62.0%; risk class I), whereas in 38 (26.8%) short hospitalization (risk class II) and in 16 (11.3%) hospitalization (risk class III) would be recommended based on CURB65-A (Figure 6). Conversely, in 218 (17.8%) and 496 (40.8%) of the 1217 hospitalized patients, outpatient treatment or a short hospitalization would be recommended according to CURB65-A risk.

## Discussion

Combining CURB65 classes with the prognostic information of ProADM in a new risk classification (CURB65-A score) offers improved risk prediction with regard to both adverse events and mortality in CAP and non-CAP-LRTI patients. This could lead to reclassification of alleged low-risk patients based on the CURB65 classification into higher risk classes and consecutive inpatient treatment; at the same time, almost 20% of patients who were treated as inpatients would be considered low risk based on CURB65-A and outpatient treatment would be strongly recommended. Thus, the results of his study suggest that the CURB65-A score can efficiently and safely increase the proportion of outpatient treatment in all LRTI patients, CAP and non-CAP.

Both PSI and CURB65 are recommended by guidelines for site of care decision in patients with CAP [29]. However, both scores were initially developed to predict mortality [3,5,30], while daily decisions have to take into account other adverse outcomes, in addition to comorbidities and non-medical factors. In the ProHOSP study, fear of adverse medical outcome was one of the most important perceived reasons by healthcare providers leading to hospital admission. This was true independent of medical risks as assessed with clinical risk scores [31]. In the ProHOSP study, 8.7% patients with CURB65 score 0-1 had an adverse event and 0.9% died, which may be considered unacceptably high by many physicians and patients to recommend or accept outpatient treatment. Therefore, in reality, triage decisions in CAP patients are not always based on these clinical risk scores [32] and are not even evidence- or guideline-based for non-CAP-LRTI. Obvious advantages of outpatient treatment include a reduced risk of health-care associated infections [33] and other nosocomial complications such as falls or delirium of elderly patients [34]. Outpatient treatment is preferred by most patients [35], improves patient and relatives' satisfaction [36], and would importantly free scarce resources in many healthcare settings, which face considerable bed shortages during the respiratory season [37]. In addition, inpatient treatment for LRTI is 8-20 times more expensive than outpatient treatment [24,36,38]. Marrie et al. [39] demonstrated that 13.5% of CAP patients with high risk based on a PSI level of IV or V, could be safely treated as outpatients with only 0.6% of those requiring ultimate hospital admission and a 0.6% mortality.

Thus, modified and improved triage algorithms are necessary [40], and new clinical pathways have been proposed [41,42]. Both of these studies were performed in Canada and little data is available on the generalizability and routine applicability of these complex pathways. Yealy et al. [43] used graded-intensity level measures to improve guideline utilization in a trial-setting in US emergency departments. However, the necessary efforts raise doubt about the feasibility in daily routine.

In contrast, readily available biomarkers which are easy to measure and quantify, might provide objective and dynamic measurements supporting clinical judgment. In addition, such biomarkers may provide an added level of confidence for physicians to actually follow recommendations of a scoring system. For prognostic purposes, ProADM is superior to PCT and other biomarkers as shown by our group and others [10,11,13,15]. These findings were largely confirmed in a multicenter US study enrolling 1653 patients with CAP, even though in the US study ProADM only added prognostic value in patients with severe CAP (PSI classes IV&V, CURB65 groups 2&3), while mortality in less severe CAP (PSI classes I-III, CURB65 group 1) was low irrespectively of ProADM values [14]. Our data confirm that ProADM significantly improves the CURB65 score for prediction of adverse events and in mortality in the high CURB65 classes. Similarly, in our study patients with low CURB65 classes (0-1) had low mortality with ProADM tertiles providing no additional information *quoad vitam*. However, increasing ProADM tertiles were associated with significantly increased risk of adverse events even in the low risk CURB65 classes.

Taken together, combining ProADM values with CURB65 scores allows a more objective triage of patients with LRTI presenting to the emergency department. It improved the prediction of adverse events in low CURB65 classes which might increase physicians' compliance with triage decisions and their acceptance by patients. Adherence to the combined risk score CURB65-A would also improve the safety of patients who formerly would have been discharged based on a low CURB65 score but in fact carried an unacceptably high risk of complications. Overall, the CURB65-A has great potential to increase the number of secure outpatient treatments by improved triage confidence and compliance.

Another strength of this study is its design as a pre-planned substudy of the randomized controlled multicenter ProHOSP intervention trial. Since it involved patients in large and medium-sized academic and non-academic hospitals, the results will be largely generalizable to many settings. This study provides evidence that CURB65 performed similarly for outcome prediction in CAP and non-CAP-LRTI patients, as recently reported [6], importantly extending their clinical utility.

There are limitations of our study. In line with the original ProHOSP study [20], we used a composite of adverse events defined as all-cause mortality, ICU admission, or any disease specific complications (i.e. empyema in all cases) as our primary endpoint. While this was done to incorporate clinically meaningful outcomes in light of sample size considerations, there are disadvantages of composite endpoints [44], such as differences in the importance of each part of the composite. One may argue that while all-cause mortality is an objective endpoint, ICU admission depends in part on the experience of the physician in charge, critical-care bed availability and other "soft" factors; however, we displayed severity criteria within ProHOSP in order to standardize ICU admission. In addition, all adverse events were monitored by an independent data safety and monitoring board. Still, because a high CURB65 score was one of the severity criteria which prompted physicians to consider admitting patients to the ICU, its prognostic performance may be artificially improved. As a consequence, this would imply a rather conservative bias in regard of the prognostic performance of ProADM. In analogy to existing risk scores and guideline recommendations, we only included medical risk factors. However, for real-life triage decisions, social, societal, organizational, functional factors and preferences of patients and relatives also have to be taken into account. In some settings these non-medical factors might be even more important and lead to reduced effectiveness of the algorithm. We are currently planning an intervention study with a multidisciplinary risk assessment including these factors to assess the efficacy of the CURB65-A score and the real-life effectiveness of the interdisciplinary triage algorithm. Finally, since the number of patients was higher in the CAP than in the non-CAP-LRTI group and since our primary endpoint was mainly driven by the outcomes in the CAP group with relatively low mortality and adverse event rates in the non-CAP-LRTI group, the utility of the CURB65-A score for patients with non-CAP-LRTIs is less certain. The similar results of the algorithm in patients with and without CAP, however, are reassuring. Nonetheless, this heterogeneous but clinically important group of non-CAP-LRTI patients deserves particular attention in future studies testing the CURB65-A score.

## Conclusions

In conclusion, the combination of ProADM and CURB65 in a novel risk score (CURB65-A) showed improved performance for triaging patients with regard to expected risks for mortality and adverse outcomes compared to CURB65 alone. Whether the theoretical benefits will actually translate into real-life improvements with improved safety by hospitalizing patients at high risk and a greater effectiveness by recommending ambulatory treatment for patients with LRTI at low risk has to be answered by future intervention studies.

## Abbreviation list

AUC: Area under the curve; CAP: community-acquired pneumonia; COPD: chronic obstructive pulmonary disease; GOLD: Global Initiative for Chronic Obstructive Lung Disease; ICU: intensive care unit; LRTI: lower respiratory tract infection; ProADM: proadrenomedullin; PSI: pneumonia severity index; ROC: receiver operating characteristic curve

## Competing interests

No commercial sponsor had any involvement in design and conduct of this study, namely, the collection, management, analysis, and interpretation of the data; and preparation, decision to submit, review, or approval of the manuscript. WA, MCC, BM and PS received support from BRAHMS to attend meetings and fulfilled speaking engagements. BM has served as a consultant to and received research support from BRAHMS. WA and BM received support from BioMérieux to attend meetings.

## Authors' contributions

WA, PS, and BM had the idea and initiated this analysis. The statistical analyses were performed by WA and PS. WA, BM and PS drafted the manuscript. All authors amended and commented on the manuscript and approved the final version.

## Pre-publication history

The pre-publication history for this paper can be accessed here:

http://www.biomedcentral.com/1471-2334/11/112/prepub
